# Supplementary material for: Lawful kinematics link eye movements to the limits of high-speed perception
Source: Nat Commun. 2025 May 8;16:3962. doi: 10.1038/s41467-025-58659-9 (PMC12062296; doi:10.1038/s41467-025-58659-9)
Supplement: Supplementary file 1 — Supplementary Information [file 41467_2025_58659_MOESM1_ESM.pdf]

Supplementary Information

Supplementary Methods: Faithful rendering of high-speed motion

The current study relied on high-speed projections using a projector that enables frame rates of 1440 Hz. This high frame rate was necessary to move stimuli at or above the speed of saccadic eye movements. The range of speeds that can be displayed in smooth quasi-continuous motion without aliasing is a function of the stimulus' spatial frequency: The stimulus must not shift more than by half a cycle of its spatial frequency. The frame rate of our projection system (1440 Hz) allowed for speeds of up to 720 dva/s at a spatial frequency of 1 cpd (Figure S1a). Higher and lower speeds could be achieved for lower and higher SFs, respectively, shaping the speed gamut of our display. For comparison, speed would have been limited to 60 dva/s for a 1 cpd grating, at a conventional frame rate of 120 Hz, resulting in a much more confined speed gamut (Figure S1b). For illustration, the maximum stimulus speed used in Experiment 1 was 442.8 dva/s, which shifted the stimulus by 0.31 phases (i.e., 0.31 dva for a spatial frequency of 1 cpd) for a total of 39 frames during the 27.1 ms movement duration. On a 120 Hz screen, the same speed and amplitude combination would have shifted the 1 cpd stimulus by 3.69 phases (i.e., 3.69 dva for a spatial frequency of 1 cpd), effectively a succession of five still frames during the 27.1 ms of stimulus motion.

Recent studies have indeed shown that high temporal resolution of displays also significantly improves human motion perception, both subjectively by reducing image jerkiness and blur[1–4] and objectively by increasing performance in motion-related visual tasks[5] and visual responses in electrophysiological brain measurements[6].

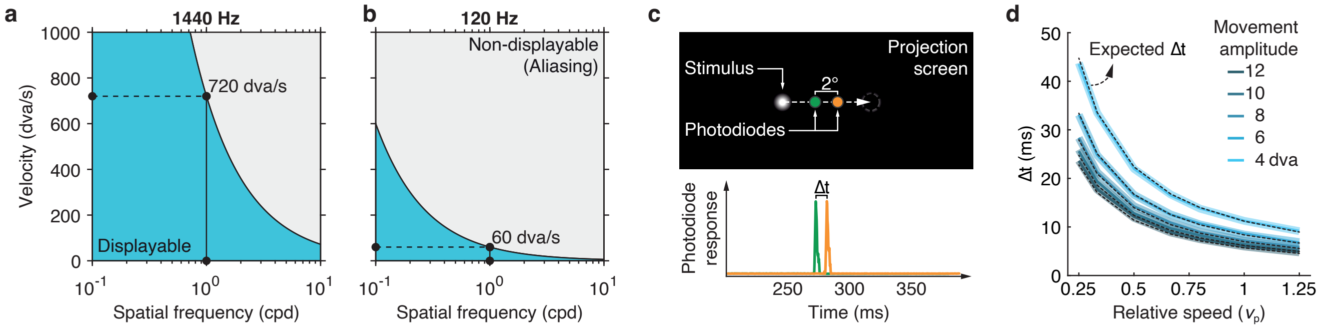

Figure S1 | Displaying motion faithfully at high speeds. Combinations of speeds and spatial frequencies that can be displayed without aliasing (blue shaded area) on **a** the 1440 Hz projector used in the experiments reported here and **b** a regular 120 Hz display. **c** Setup for stimulus speed measurements using two photodiodes. We measured the time between the maximum response of two photodiodes (Δt) placed 2 dva apart and compared it to the expected Δt for a 2 dva movement given a specified stimulus speed. **d** Δt as a function of movement amplitude, speed, and direction. Transparent bold lines and opaque thin lines (which overlap perfectly for each amplitude condition) indicate leftward and rightward motion, respectively. Standard deviations of Δt (not displayed) were below 1 ms for every condition. Dotted black lines indicate Δt as expected for a given condition.

We performed photodiode measurements to ensure accurate timing of the 1440 Hz projection and to ascertain that the physical speed of stimuli corresponded to our specifications. We used a LM03 light meter (Cambridge Research Systems Ltd., Rochester, UK), a device previously applied to evaluate the timing of computer monitors[7,8], to record one second of luminance measurements from two photodiodes at 4000 Hz. We aligned the two photodiodes horizontally, each with a distance of 1 dva from the screen center (Figure S1c), resulting in a distance of 2 dva between the two sensors. We collected data from 1427 trials, in which a white Gaussian blob on black background traveled across the two sensors with varying movement amplitudes and relative movement speeds (v_{rel} = {1/4, 1/3, 1/2, 2/3, 4/5, 1, 5/4}) as used in our experiments. As speed was fixed during each trial, the time passed between the two photodiode responses directly indicated the speed at which the stimulus traveled across the screen. Physical stimulus speed was indeed exactly as specified for both leftward and rightward motion directions, and across all movement amplitudes (Figure S1d). Minimal code to drive the LM03 light meter, as well as data and analyses of the above described measurements are provided at `https://github.com/richardschweitzer/LM03_lightmeter`.

Supplementary Note 1: Results of statistical models (Exp. 1)

Table S1 | Mode and 95% *CI*s (in $\log_{10}$-units) for parameter estimates in Experiment 1 when reparameterizing thresholds as orthogonal contrasts (N = 10 observers). Analyses are shown separately for absolute speed, relative speed, absolute duration, and relative duration of the movement. Asterisks indicate *CI*s that do not include zero.

	Contrast	Mode	$\mathrm{CI}_{0.025}$	$\mathrm{CI}_{0.975}$	
Absolute speed					
Amplitude	Intercept	2.166	2.139	2.193	*
	Linear	0.584	0.514	0.651	*
	Quadratic	-0.185	-0.237	-0.141	*
	Cubic	0.042	0.008	0.085	*
	Quartic	-0.070	-0.166	0.049	
Relative speed					
Amplitude	Intercept	-0.278	-0.306	-0.251	*
	Linear	-0.059	-0.126	0.007	
	Quadratic	-0.002	-0.050	0.054	
	Cubic	0.006	-0.030	0.046	
	Quartic	-0.048	-0.136	0.061	
Absolute duration					
Amplitude	Intercept	1.706	1.679	1.733	*
	Linear	0.592	0.527	0.661	*
	Quadratic	-0.030	-0.079	0.020	
	Cubic	-0.005	-0.048	0.023	
	Quartic	0.065	-0.056	0.150	
Relative duration					
Amplitude	Intercept	-0.104	-0.122	-0.086	*
	Linear	-0.031	-0.074	0.009	
	Quadratic	0.006	-0.029	0.038	
	Cubic	0.004	-0.021	0.031	
	Quartic	-0.018	-0.093	0.066	

Supplementary Note 2: Visibility thresholds for detection of high-speed motion (Exp. 2a)

In a variant of Experiment 1, we eliminated the vertical component of the stimulus' movement trajectory such that it followed a straight horizontal path (Figure S2a) while maintaining the same range of horizontal speeds (Figure S2b-c). We manipulated the presence of continuous, horizontal motion. In motion-absent trials (50%), the stimulus was blanked for the movement duration *D* and displaced to its new location. Observers were asked to distinguish motion-present from motion-absent trials, providing us with a measure of visibility in which the curvature of the path was eliminated. All other aspects of the task were identical to Experiment 1.

For each observer, we calculated visual sensitivity as $d' = z(\mathrm{HR}) - z(\mathrm{FAR})$, that is, the difference of z-transformed hit rates (HR) and false alarm rates (FAR) for each combination of movement amplitude and speed. We then fitted (nonlinear least-squares) the mean across observers with a Naka-Rushton function,

$$d'_A = d'_{max} \frac{v^n}{v^n + v_{50}^n} \tag{1}$$

where d'_A is sensitivity as a function of movement amplitude *A*, d'_{max} is the asymptotic performance at low speeds, *n* reflects the slope, and v_{50} is the speed at half the asymptotic performance, that is, the visibility threshold.

In this detection task, performance was generally lower than in the discrimination task (Exp. 1), but showed the same dependence on movement amplitude and speed. Expressed as a function of *absolute* stimulus speed (Figure S2d, *left*), performance systematically increased with movement amplitude (fixed-effect estimates of a linear mixed effects model with random effects for intercept β_0 and slope β: β_0 = 53.64 dva/s, $t(48) = 9.08$, $p < 0.001$; β = 6.12 s^{-1}, $t(48) = 12.60$, $p < 0.001$), shifting psychometric functions systematically to the right. Visibility thresholds (Figure S2d, *right*) closely followed the prediction based on the main sequence (solid white lines), with remarkable consistency across observers (gray lines). Expressed as a function of *relative* movement speed (Figure S2e, *left*), psychometric functions collapsed, such that thresholds settled around 36% of saccadic peak velocity (Figure S2e, right) with little to no variation across movement amplitudes (β_0 = 0.389 v_p, $t(48) = 13.82$, $p < 0.001$; β = -0.003 v_p/dva, $t(48) = -1.63$, $p = 0.109$). Again this confirmed the prediction based on the main-sequence relation of saccades.

These results show that the vertical component of movement trajectories in our discrimination task was not a critical factor for the pattern of results in Experiment 1. The fact that observers' sensitivity collapsed to zero at high speeds highlights that continuous motion was indeed phenomenologically indistinguishable from apparent motion.

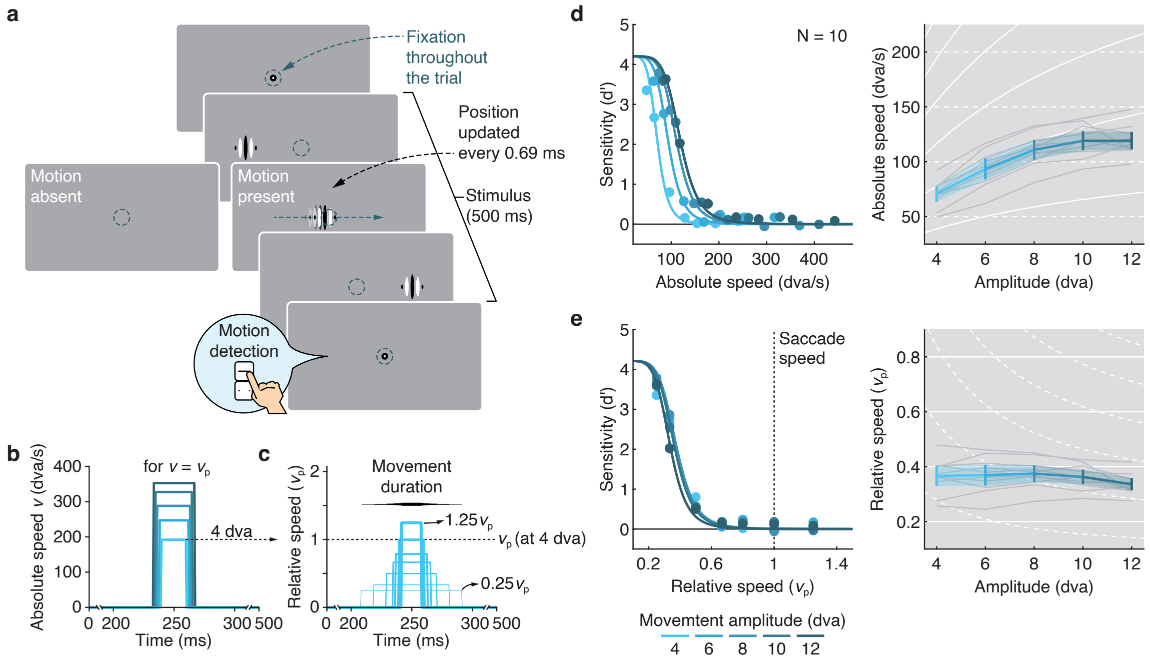

Figure S2 | Motion detection thresholds for constant velocity profiles (Exp. 2a). **a** Trial procedure. Observers (*N* = 10) judged whether the stimulus jumped (Motion absent; stimulus blanked) or moved continuously on a straight horizontal path (Motion present) from its initial to its final location. **b** Absolute speed for each movement amplitude for relative speed of 1. **c** Relative movement speeds for 4 dva amplitude. **d-e** Performance (left) and visibility thresholds (v_{50}, right) obtained from best-fitting Naka-Rushton functions as a function of absolute and relative movement speed, respectively, plotted for each amplitude. Conventions as in Figure 3a and b.

Supplementary Note 3: Visibility thresholds for detection of high-speed motion with saccadic velocity profiles (Exp. 2b)

In a variant of Experiment 2a (Figure S3a), we varied the speed of the stimulus over time to mimic the velocity profile of a saccade (Figure S3b; see Methods). We increased the range of relative movement speeds up to twice the saccadic peak velocity, as we expected higher performance from time-varying velocity profiles. As in Experiment 2a, participants reported whether the stimulus moved continuously (motion present) or instead, jumped (motion absent) from its initial to its final location. To prevent that observers used sudden onsets and offsets to establish the absence of motion, we used the velocity profile of the motion-present condition for a contrast ramp (see Methods for details).

It is important to note that perceptual omission during active saccades is imperfect due to slow velocities during the acceleration and deceleration phases of the movement[8], and the duration of these phases depend on movement amplitude[9]. We thus predicted thresholds to depend moderately on movement amplitude, but in a way that is inconsistent with a fixed velocity threshold. Indeed, while on average performance was higher compared to a constant velocity profile (Supplementary Note 2), it showed a clear dependence on movement distance and speed. Expressed as a function of *absolute* stimulus speed (Figure S3d, *left*), performance systematically increased with movement amplitude (fixed-effect estimates of a linear mixed effects model with random effects for intercept β_0 and slope β: β_0 = 25.98 dva/s, $t(48)$ = 2.73, p = 0.009; β = 22.56 s^{-1}, $t(48)$ = 10.78, p < 0.001), shifting psychometric functions systematically to the right. Visibility thresholds (Figure S3d, *right*) again closely followed the prediction based on the main sequence (solid white lines), with slightly steeper slopes and remarkable consistency across observers (gray lines). Expressed as a function of *relative* movement speed (Figure S3e, *left*), the distance between psychometric functions decreased markedly, and thresholds settled around 60 to 80% of saccadic peak velocity (Figure S3e, right). As predicted, there remained a small but significant increase with movement amplitude (β_0 = 0.456 v_p, $t(48)$ = 6.67, p < 0.001; β = -0.03 v_p/dva, $t(48)$ = 7.64, p < 0.001), which was inconsistent with the prediction of a constant velocity threshold (*dashed white lines*).

These results confirm that visibility thresholds systematically depend on movement amplitude in a way well-described by the main-sequence relation of saccadic eye movements. Moreover, they suggest that our key findings are relevant to saccadic omission[10]: Perceptual omission from time-varying velocity profiles is consistent with omission experienced during active saccades[8].

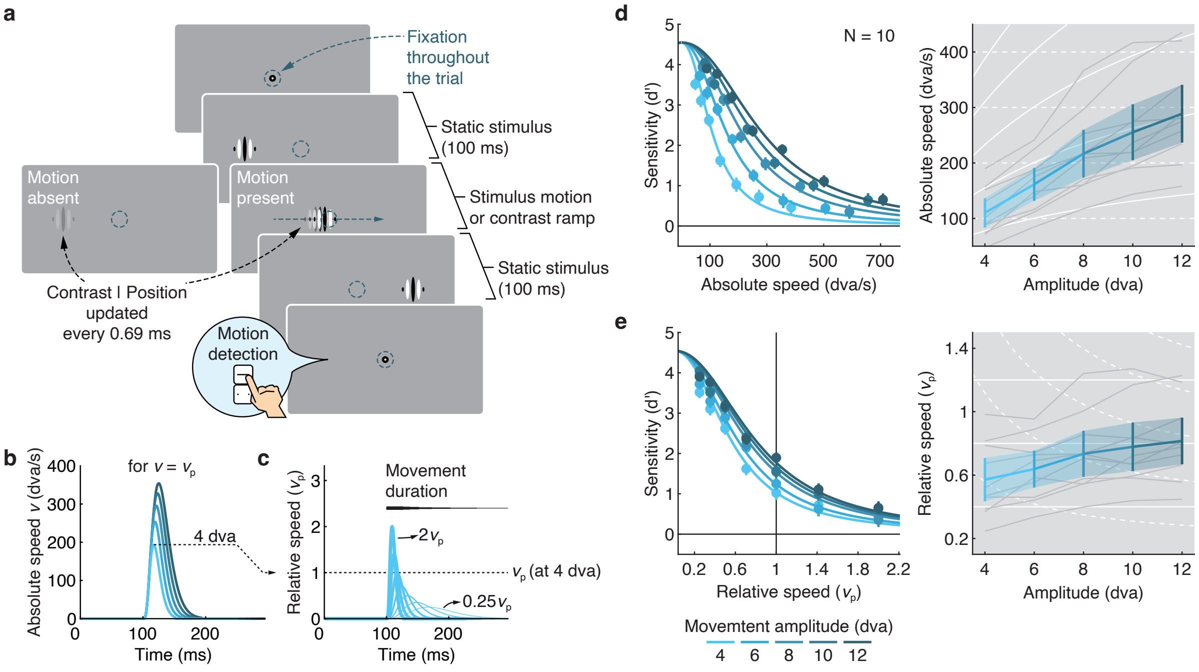

Figure S3 | Motion detection thresholds for saccadic velocity profiles (Exp. 2b). **a** Trial procedure. Observers (N = 10) judged whether the stimulus jumped (Motion absent; contrast ramped down to zero contrast, jumped to its new location before ramping up to full contrast again) or moved continuously on a straight horizontal path (Motion present) from its initial to its final location. **b** Absolute speed for each movement amplitude for relative speed of 1. **c** Relative speeds for 4 dva amplitude. **d-e** Performance (left) and visibility thresholds (v_{50}; right) obtained from best-fitting Naka-Rushton functions as a function of absolute and relative speed, respectively, plotted for each amplitude. Conventions as in Figure 3a and b.

Supplementary Note 4: Variability in visibility thresholds and saccade kinematics (Exp. 3 and 4)

Experiments 3 and 4 extended our protocol from horizontal to vertical motion. Experiment 3 showed that, for each movement direction, performance depended on a conjunction of movement amplitude, speed, and duration (Figure S4), such that visibility thresholds were well predicted by the standard main-sequence relations for peak velocity (Figure S4a) and duration (Figure S4b). Visibility thresholds for absolute and relative speed were higher for rightward compared to leftward and for downward compared to upward motion (Table S2 and Table S3). Similarly, visibility thresholds for absolute and relative duration were lower for rightward compared to leftward and for downward compared to upward motion (Table S4 and Table S5). These threshold differences between movement directions were consistent across observers and replicated in Experiment 4 with a larger sample tested in a single movement amplitude (8 dva; Figure S4a,b).

For each participant in Experiments 3 and 4, we also recorded visually-guided saccades (4 to 12 dva) in separate blocks of trials, and estimated each individual's retinal motion kinematics during saccades (see Methods, *Analysis of saccade kinematics*). Specifically, we fitted all saccades' velocity profiles with a biophysical model that isolated the relevant eyeball velocity from the recorded pupil velocity[11,12]. Movement speed and duration related tightly to saccade amplitude, defining the main-sequence. We thus fitted each individuals' eyeball kinematics (peak velocity and saccade duration), separately for each movement direction, with functions that capture the respective main-sequence relation. These relations varied both across individuals and, within individuals, across saccade directions (Figure S4c,d). Specifically, across both experiments, and consistent with previous findings[13–15], upward saccades compared to downwards saccades were faster (Exp. 3: $t(5) = 5.13$, $p = 0.004$; Exp. 4: $t(35) = 5.44$, $p < 0.001$) and of shorter duration (Exp. 3: $t(5) = -4.63$, $p = 0.006$; Exp. 4: $t(35) = -4.94$, $p < 0.001$), while leftward and rightward saccades did not differ in their peak velocities and durations (all $ps > 0.21$).

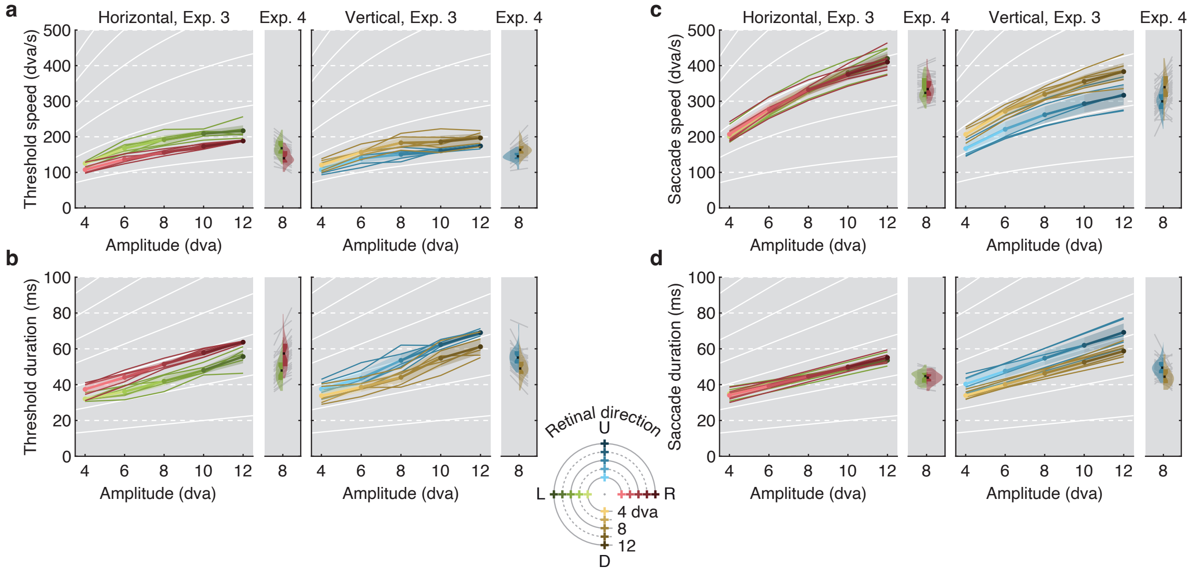

Figure S4 | Visibility thresholds and saccadic main sequence in Experiments 3 and 4 as a function of movement amplitude and retinal direction. Visibility thresholds were obtained from psychometric functions fitted to observers' performance as a function of **a** absolute movement speed, **b** absolute movement duration (analogous to Figure 3a,c). For each movement direction (color coded), the modes of estimated threshold parameters of the psychometric functions are shown as a function of movement amplitude. **c** Saccade speed and **d** saccade duration are shown as a function of movement amplitude and retinal direction. To allow for direct comparison of visibility thresholds and saccade data, color codes refer to the movement's retinal direction (i.e., for saccades this is the direction opposite of the saccade's spatial direction, corresponding to the motion direction of of the retinal image during the saccade). For Experiment 3, values averaged across observers (N = 6; thick opaque lines) are shown along with each individual's data (thin transparent lines). Error bands are 95% credible intervals. For Experiment 4, violin plots show distributions of individual data (N = 36; gray lines). Solid white lines in the background indicate predictions in which thresholds depend on movement amplitude, proportional to the standard main sequence; dashed white lines indicate predictions in which thresholds are independent of movement amplitude.

Table S2 | Mode and 95% *CI*s (in $\log_{10}$-units) for parameter estimates in Experiment 3 when reparameterizing absolute-speed thresholds as orthogonal contrasts (*N* = 6 observers). For directon conditions R, L, U, and D correspond to rightward, leftward, upward, and upward retinal motion, respectively. Asterisks depict coefficients for which the *CI* does not include zero.

	Contrast	Mode	$CI_{0.025}$	$CI_{0.975}$	
Intercept		2.198	2.150	2.243	*
Direction	LR – DU	-0.288	-0.704	0.128	
	L – R	0.380	0.264	0.505	*
	D – U	-0.342	-0.429	-0.247	*
Direction	Linear	0.586	0.465	0.716	*
(Rightward)	Quadratic	-0.135	-0.226	-0.061	*
	Cubic	0.045	-0.019	0.108	
	Quartic	-0.010	-0.220	0.184	
Direction	Linear	0.569	0.499	0.647	*
(Leftward)	Quadratic	-0.238	-0.330	-0.140	*
	Cubic	0.043	-0.084	0.175	
	Quartic	0.002	-0.226	0.163	
Direction	Linear	0.496	0.302	0.707	*
(Downward)	Quadratic	-0.234	-0.339	-0.145	*
	Cubic	0.076	-0.037	0.195	
	Quartic	0.059	-0.141	0.309	
Direction	Linear	0.436	0.264	0.623	*
(Upward)	Quadratic	-0.184	-0.290	-0.071	*
	Cubic	0.106	0.020	0.178	*
	Quartic	-0.041	-0.267	0.214	

Table S3 | Mode and 95% *CI*s (in $\log_{10}$-units) for parameter estimates in Experiment 3 when reparameterizing relative-speed thresholds as orthogonal contrasts (*N* = 6 observers). For directon conditions R, L, U, and D correspond to rightward, leftward, upward, and upward retinal motion, respectively. Asterisks depict coefficients for which the *CI* does not include zero.

	Contrast	Mode	$CI_{0.025}$	$CI_{0.975}$	
Intercept		-0.248	-0.292	-0.200	*
Direction	LR – DU	-0.285	-0.709	0.135	
	L – R	0.382	0.264	0.503	*
	D – U	-0.343	-0.432	-0.243	*
Direction	Linear	-0.051	-0.181	0.073	
(Rightward)	Quadratic	0.039	-0.026	0.130	
	Cubic	0.004	-0.059	0.071	
	Quartic	-0.013	-0.205	0.205	
Direction	Linear	-0.063	-0.140	0.006	
(Leftward)	Quadratic	-0.050	-0.131	0.043	
	Cubic	0.002	-0.121	0.127	
	Quartic	0.009	-0.196	0.177	
Direction	Linear	-0.140	-0.343	0.067	
(Downward)	Quadratic	-0.048	-0.148	0.043	
	Cubic	0.035	-0.082	0.153	
	Quartic	0.110	-0.121	0.332	
Direction	Linear	-0.200	-0.374	-0.018	*
(Upward)	Quadratic	0.008	-0.098	0.112	
	Cubic	0.061	-0.026	0.151	
	Quartic	-0.030	-0.256	0.227	

Table S4 | Mode and 95% *CI*s (in $\log_{10}$-units) for parameter estimates in Experiment 3 when reparameterizing absolute-duration thresholds as orthogonal contrasts (*N* = 6 observers). For directon conditions R, L, U, and D correspond to rightward, leftward, upward, and upward retinal motion, respectively. Asterisks depict coefficients for which the *CI* does not include zero.

	Contrast			Mode	$CI_{0.025}$	$CI_{0.975}$	
Intercept				1.675	1.627	1.721	*
Direction	LR	–	DU	0.289	-0.145	0.716	
	L	–	R	-0.380	-0.501	-0.265	*
	D	–	U	0.341	0.250	0.428	*
Direction	Linear			0.590	0.461	0.721	*
(Rightward)	Quadratic			-0.072	-0.161	-0.004	*
	Cubic			-0.008	-0.072	0.055	
	Quartic			0.009	-0.201	0.214	
Direction	Linear			0.609	0.529	0.683	*
(Leftward)	Quadratic			0.010	-0.079	0.105	
	Cubic			-0.016	-0.126	0.112	
	Quartic			0.018	-0.185	0.205	
Direction	Linear			0.673	0.468	0.875	*
(Downward)	Quadratic			0.015	-0.077	0.124	
	Cubic			-0.043	-0.156	0.072	
	Quartic			-0.087	-0.309	0.114	
Direction	Linear			0.736	0.546	0.909	*
(Upward)	Quadratic			-0.046	-0.147	0.058	
	Cubic			-0.061	-0.150	0.018	
	Quartic			0.032	-0.223	0.264	

Table S5 | Mode and 95% *CI*s (in $\log_{10}$-units) for parameter estimates in Experiment 3 when reparameterizing relative-duration thresholds as orthogonal contrasts (*N* = 6 observers). For directon conditions R, L, U, and D correspond to rightward, leftward, upward, and upward retinal motion, respectively. Asterisks depict coefficients for which the *CI* does not include zero.

	Contrast			Mode	$CI_{0.025}$	$CI_{0.975}$	
Intercept				-0.076	-0.110	-0.042	*
Direction	LR	–	DU	-0.227	-0.507	0.091	
	L	–	R	0.325	0.202	0.458	*
	D	–	U	-0.260	-0.332	-0.180	*
Direction	Linear			-0.026	-0.098	0.047	
(Rightward)	Quadratic			0.035	-0.024	0.085	
	Cubic			-0.001	-0.045	0.046	
	Quartic			0.011	-0.122	0.139	
Direction	Linear			-0.075	-0.152	0.005	
(Leftward)	Quadratic			-0.046	-0.140	0.044	
	Cubic			0.014	-0.099	0.138	
	Quartic			0.010	-0.208	0.193	
Direction	Linear			-0.132	-0.310	0.053	
(Downward)	Quadratic			-0.016	-0.111	0.046	
	Cubic			0.039	-0.054	0.123	
	Quartic			0.121	-0.054	0.286	
Direction	Linear			-0.120	-0.228	-0.009	*
(Upward)	Quadratic			0.003	-0.066	0.060	
	Cubic			0.038	-0.014	0.087	
	Quartic			-0.008	-0.151	0.141	

Supplementary Note 5: Stepwise regressions (Exp. 3 and 4)

Saccade amplitude (manipulated in Exp. 3, but not in Exp. 4), saccade direction, and individual differences in saccade kinematics each contribute to the speed of visual motion that saccades impose on the retina (i.e., retinal speed, in the opposite direction of the saccade itself). To isolate the unique contributions of these variables to the variation in visibility thresholds, we performed stepwise hierarchical regressions and included predictors (direction coded as a categorical variable with leftward as a reference) in order of ΔBIC magnitude (reduction in Bayesian Information Criterion) when included individually in the model.

In Experiment 3, when included individually, all variables explained variance in speed thresholds (Direction: ΔBIC = -8.24, Amplitude: ΔBIC = -114.43; Retinal speed: ΔBIC = -130.00). Stepwise inclusion showed that unique variance in speed thresholds (Figure 5a, *left*) was explained by each predictor, individual differences in retinal speed (β = 0.14, *SE* = 0.05, *t* = 2.81, p = 0.006), saccade amplitude (β = 6.15, *SE* = 1.28, *t* = 4.81, p < 0.001) and direction (Right: β = -29.63, *SE* = 3.86, *t* = -7.68, p < 0.001; Up: β = -25.82, *SE* = 5.22, *t* = -4.94, p < 0.001; Down: β = -11.54, *SE* = 3.92, *t* = -2.94, p < 0.001), capturing a total of 81.4% of variance in visibility thresholds (*F* = 99.9, *p* < 0.001). As an alternative model, we set up the regression using individual differences in spatial speed (i.e., the direction of the saccade was the same as the direction of motion) instead of retinal speed as a predictor and compared this model to the model including retinal speed. The model using retinal speed was 12.8 times more likely to account for the data ($\log(BF_{10})$ = 12.8; ΔBIC_{10} = -58.82). Finally, we repeated this regression predicting thresholds from the individual differences in speed ±90°away from saccade direction, again confirming the superiority of the regression based on retinal direction (+90°: $\log(BF_{10})$ = 19.3; ΔBIC_{10} = -88.83; −90°: $\log(BF_{10})$ = 9.2; ΔBIC_{10} = -42.58).

Knowing from Experiment 1 that amplitude relates to speed in similar ways for visibility thresholds as for saccades, we expected large correlations between saccade kinematics and visibility thresholds in Experiment 3. The regression analysis, however, shows that individual and direction-specific differences in the saccadic main sequence are predicted specifically by the retinal direction of saccades, and reflected in visibility thresholds above and beyond the general amplitude-speed relation.

Experiment 4 replicated these results, notably in the absence of an amplitude manipulation, thus controlling for the impact of amplitude on speed experimentally rather than statistically. Here, both saccade direction and retinal speed explained variance in visibility thresholds when included individually (Direction: ΔBIC = -27.28, Retinal speed: ΔBIC = -13.35). Indeed, retinal speed (β = 0.18, *SE* = 0.05, *t* = 3.95, p < 0.001) and saccade direction (Right: β = -28.24, *SE* = 4.69, *t* = -6.02, p < 0.001; Up: β = -18.44, *SE* = 4.98, *t* = -3.70, p < 0.001; Down: β = -5.76, *SE* = 4.69, *t* = -1.23, p = 0.222) explained unique variance, yielding a total of 32.9% of variance explained (*F* = 17.1, *p* < 0.001). As before, we repeated the regressions using individual differences in spatial speed as well as the speed of saccades ±90°away from saccade direction, by and large supporting the superiority of the regression model based on retinal direction (spatial: $\log(BF_{10})$ = 3.5; ΔBIC_{10} = -15.97; +90°: $\log(BF_{10})$ = 4.0; ΔBIC_{10} = -18.25; −90°: $\log(BF_{10})$ = 0.7; ΔBIC_{10} = -3.31).

Using the same approach as for speed thresholds, we predicted duration thresholds (Figure 5b). In Experiment 3, all variables explained variance in duration thresholds when included individually (Direction: ΔBIC = -1.88, Amplitude: ΔBIC = -164.86; Retinal speed: ΔBIC = -143.64), but only saccade amplitude (β = 3.48, *SE* = 0.13, *t* = 27.32, p < 0.001) and direction (Right: β = -8.13, *SE* = 1.02, *t* = -11.97, p < 0.001; Up: β = -10.45, *SE* = 1.02, *t* = -10.26, p < 0.001; Down: β = -3.73, *SE* = 1.02, *t* = -3.66, p < 0.001) explained unique variance, capturing a total of 88.3% of variance in duration thresholds (*F* = 99.9, *p* < 0.001). Adding retinal speed (ΔBIC = 2.73)to the model did not explain additional variance. However, when we repeated the regressions using individual differences in spatial speed as well as the speed of saccades ±90°away from saccade direction, we found support of the superiority of the regression model based on retinal direction (spatial: $\log(BF_{10})$ = 15.3; ΔBIC_{10} = -70.35; +90°: $\log(BF_{10})$ = 21.1; ΔBIC_{10} = -97.19; −90°: $\log(BF_{10})$ = 11.9; ΔBIC_{10} = -54.75).

In Experiment 4, in the absence of an amplitude manipulation, movement direction (Direction: ΔBIC = -25.07) and retinal speed (Retinal speed: ΔBIC = -5.54) explained variance when included as individual predictors.Importantly, both saccade direction (Right: β = 9.69, *SE* = 1.68, *t* = -5.77, p < 0.001; Up: β = 5.74, *SE* = 1.84, *t* = 3.11, p = 0.002; Down: β = 1.18, *SE* = 1.68, *t* = 0.70, p = 0.482) and retinal speed (β = 0.39, *SE* = 0.14, *t* = 2.90, p = 0.004) explained unique variance in duration thresholds, yielding a total of 28.6% of variance explained (*F* = 13.9, *p* < 0.001). Once more, we repeated the regressions using individual differences in spatial speed as well as the speed of saccades ±90°away from saccade direction, by and large supporting the superiority of the regression model based on retinal direction (spatial: $\log(BF_{10})$ = 2.1; ΔBIC_{10} = -9.87; +90°: $\log(BF_{10})$ = 2.2; ΔBIC_{10} = -10.26; −90°: $\log(BF_{10})$ = 0.0; ΔBIC_{10} = -0.09).

Thus, across both experiments, we found that individual saccade kinematics predict visibility thresholds for high-speed stimuli presented during fixation. As predicted, these correlations were specific to the retinal as opposed to the spatial direction of saccades (results reported in main text). Even when controlling for the mediating influence of movement amplitude on these correlations—either statistically (Exp. 3) or experimentally (Exp. 4)—the retinal speed imposed by saccades explained unique variance in visibility thresholds. The only exception from this was the prediction of duration thresholds in Exp. 3, for which saccade amplitude and direction left barely any variance unexplained.

Supplementary Note 6: Results of statistical models for relative movement speed (Exp. 5)

Table S6 | Mode and 95% *CI*s (in $\log_{10}$-units) for parameter estimates in Experiment 5 when reparameterizing relative-speed thresholds as orthogonal contrasts (*N* = 11 observers). For static-endpoint duration (Stat-end-dur), conditions 1, 2, 3, and 4 correspond to 0, 12.5, 50, and 200 ms, respectively. Asterisks indicate *CI*s that do not include zero.

	Contrast	Mode	$CI_{0.025}$	$CI_{0.975}$	
Intercept		-0.248	-0.280	-0.216	*
Stat-end-dur	1 – 234	-2.140	-2.456	-1.820	*
	2 – 34	-0.279	-0.453	-0.124	*
	3 – 4	-0.078	-0.129	0.008	
Amplitude (0 ms)	Linear	-0.857	-0.942	-0.790	*
	Quadratic	0.185	0.113	0.276	*
	Cubic	-0.020	-0.084	0.036	
	Quartic	0.055	-0.072	0.178	
Amplitude (12.5 ms)	Linear	-0.166	-0.229	-0.098	*
	Quadratic	-0.058	-0.126	-0.005	*
	Cubic	-0.030	-0.080	0.028	
	Quartic	0.001	-0.147	0.144	
Amplitude (50 ms)	Linear	-0.049	-0.122	0.016	
	Quadratic	-0.024	-0.106	0.074	
	Cubic	-0.001	-0.052	0.058	
	Quartic	-0.049	-0.250	0.183	
Amplitude (200 ms)	Linear	-0.067	-0.131	0.012	
	Quadratic	-0.011	-0.107	0.085	
	Cubic	-0.058	-0.155	0.039	
	Quartic	-0.000	-0.236	0.228	

Supplementary Note 7: Results for absolute movement speed and movement duration (Exp. 5)

When describing Experiment 5 in the main text, we reported an analysis of visibility thresholds as a function of relative movement speed. Here, we also report the corresponding analyses as a function of absolute movement speed (Figure S5a; Table S7), absolute movement duration (Figure S5b; Table S8), and relative movement duration (Figure S5c; Table S9) for each movement amplitude and pre- and post-movement stimulus duration.

Table S7 | Mode and 95% *CI*s (in $\log_{10}$-units) for parameter estimates in Experiment 5 when reparameterizing absolute-speed thresholds as orthogonal contrasts (N = 11 observers). For static-endpoint duration (Stat-end-dur), conditions 1, 2, 3, and 4 correspond to 0, 12.5, 50, and 200 ms, respectively. Asterisks depict coefficients for which the *CI* does not include zero.

	Contrast	Mode	$CI_{0.025}$	$CI_{0.975}$	
Intercept		2.196	2.163	2.230	*
Stat-end-dur	1 – 234	-2.124	-2.464	-1.823	*
	2 – 34	-0.275	-0.454	-0.124	*
	3 – 4	-0.058	-0.131	0.002	
Amplitude (0 ms)	Linear	-0.229	-0.313	-0.150	*
	Quadratic	0.008	-0.073	0.084	
	Cubic	0.018	-0.049	0.074	
	Quartic	0.028	-0.094	0.174	
Amplitude (12.5 ms)	Linear	0.480	0.415	0.546	*
	Quadratic	-0.248	-0.313	-0.194	*
	Cubic	0.015	-0.036	0.066	
	Quartic	-0.011	-0.169	0.121	
Amplitude (50 ms)	Linear	0.587	0.518	0.662	*
	Quadratic	-0.205	-0.301	-0.120	*
	Cubic	0.044	-0.020	0.095	
	Quartic	-0.076	-0.274	0.155	
Amplitude (200 ms)	Linear	0.577	0.510	0.649	*
	Quadratic	-0.203	-0.292	-0.102	*
	Cubic	-0.011	-0.118	0.074	
	Quartic	-0.030	-0.248	0.209	

Table S8 | Mode and 95% *CI*s (in $\log_{10}$-units) for parameter estimates in Experiment 5 when reparameterizing absolute-duration thresholds as orthogonal contrasts (N = 11 observers). For static-endpoint duration (Stat-end-dur), conditions 1, 2, 3, and 4 correspond to 0, 12.5, 50, and 200 ms, respectively. Asterisks depict coefficients for which the *CI* does not include zero.

	Contrast	Mode	$CI_{0.025}$	$CI_{0.975}$	
Intercept		1.677	1.643	1.710	*
Stat-end-dur	1 – 234	2.146	1.827	2.453	*
	2 – 34	0.285	0.124	0.457	*
	3 – 4	0.063	0.000	0.126	*
Amplitude (0 ms)	Linear	1.404	1.326	1.484	*
	Quadratic	-0.214	-0.305	-0.145	*
	Cubic	0.019	-0.043	0.082	
	Quartic	-0.025	-0.182	0.077	
Amplitude (12.5 ms)	Linear	0.692	0.632	0.761	*
	Quadratic	0.026	-0.030	0.091	
	Cubic	0.014	-0.036	0.070	
	Quartic	-0.001	-0.128	0.151	
Amplitude (50 ms)	Linear	0.594	0.510	0.660	*
	Quadratic	-0.011	-0.108	0.076	
	Cubic	-0.014	-0.067	0.041	
	Quartic	0.062	-0.162	0.262	
Amplitude (200 ms)	Linear	0.609	0.529	0.665	*
	Quadratic	-0.014	-0.114	0.078	
	Cubic	0.050	-0.042	0.147	
	Quartic	-0.011	-0.227	0.236	

Table S9 | Mode and 95% *CI*s (in $\log_{10}$-units) for parameter estimates in Experiment 5 when reparameterizing relative-duration thresholds as orthogonal contrasts (*N* = 11 observers). For static-endpoint duration (Stat-end-dur), conditions 1, 2, 3, and 4 correspond to 0, 12.5, 50, and 200 ms, respectively. Asterisks depict coefficients for which the *CI* does not include zero.

	Contrast			Mode	$CI_{0.025}$	$CI_{0.975}$	
Intercept				-0.065	-0.091	-0.039	*
Stat-end-dur	1	–	234	-2.416	-2.873	-1.971	*
	2	–	34	-0.193	-0.314	-0.076	*
	3	–	4	-0.042	-0.082	0.000	
Amplitude (0 ms)	Linear			-1.090	-1.250	-0.950	*
	Quadratic			0.417	0.316	0.530	*
	Cubic			-0.057	-0.129	0.021	
	Quartic			-0.004	-0.141	0.148	
Amplitude (12.5 ms)	Linear			-0.111	-0.150	-0.066	*
	Quadratic			-0.044	-0.085	-0.006	*
	Cubic			-0.012	-0.053	0.025	
	Quartic			-0.009	-0.100	0.109	
Amplitude (50 ms)	Linear			-0.024	-0.077	0.030	
	Quadratic			-0.002	-0.058	0.046	
	Cubic			0.005	-0.034	0.036	
	Quartic			-0.016	-0.147	0.129	
Amplitude (200 ms)	Linear			-0.032	-0.078	0.012	
	Quadratic			-0.015	-0.064	0.048	
	Cubic			-0.025	-0.085	0.024	
	Quartic			-0.023	-0.152	0.133	

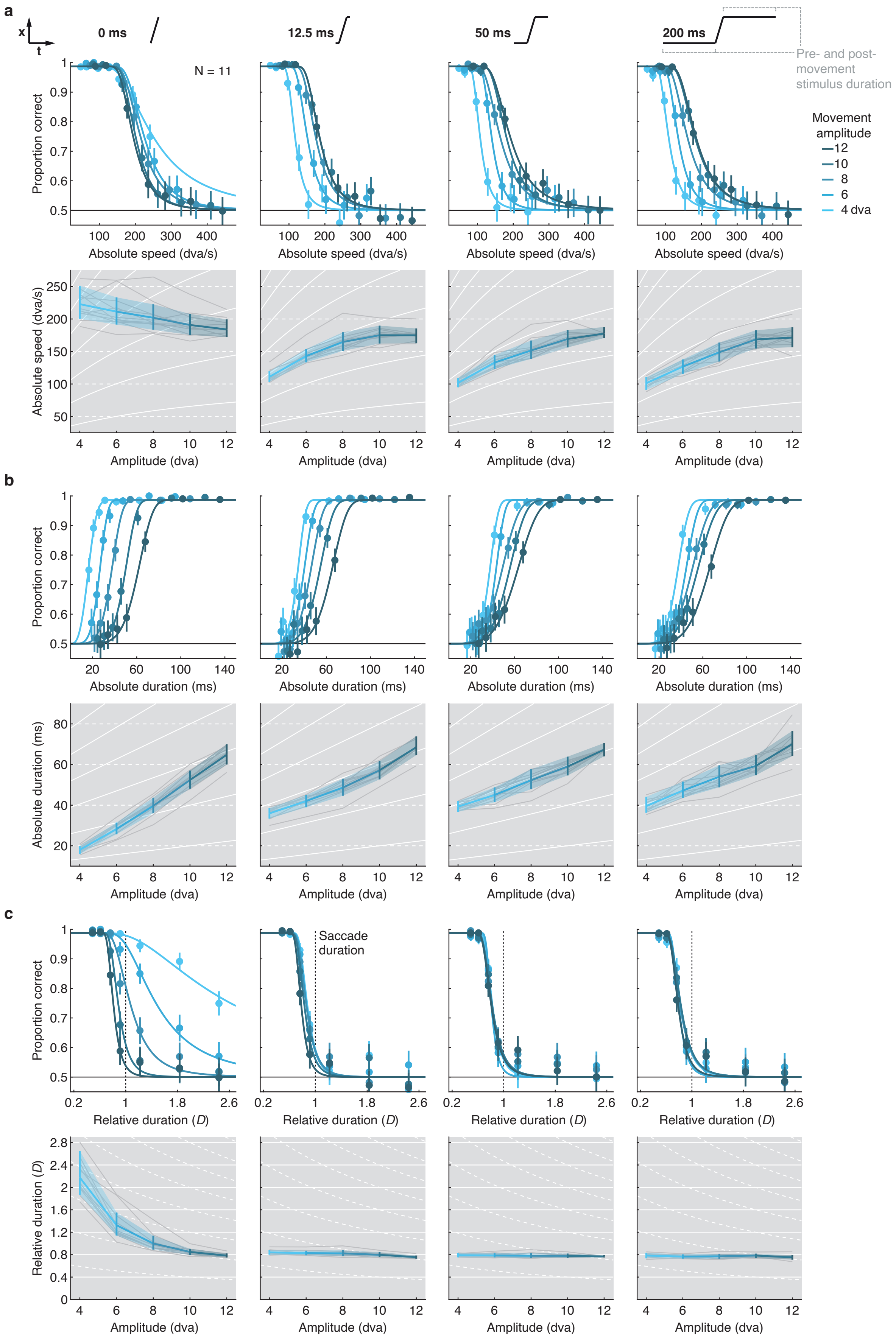

Figure S5 | Pre- and post-movement stimulus presence gives rise to main-sequence relation in motion visibility. Psychometric functions and resulting visibility thresholds, expressed as **a** absolute movement speed, **b** absolute movement duration, and **c** relative movement duration are shown as a function of movement amplitude and pre- and post-movement stimulus duration, varied between 0 ms (left), 12.5 ms (center-left), 50 ms (center-right), and 200 ms (right). Data were obtained from N = 11 observers completing a total of 57,672 trials. Conventions as in Figures 3 and 6.

Supplementary Note 8: Grid-search analyses

To explore the parameter space of the Early-vision model and to understand which combinations of parameters led to the emergence of the described model behavior, we performed a large-scale grid-search analysis across parameters σ_{RF} and θ_{RF}, that is, the standard deviation of the spatial Gaussian kernel and the scale of the temporal Gamma kernel, which were varied from 0.05 to 5 cpd and 1 to 100 ms, respectively, each with nine logarithmic steps (Figure S6a). Model simulations were run 25 times for each of the resulting 81 grid points. Code and data can be found online at `https://github.com/richardschweitzer/ModelingVisibilityOfSaccadelikeMotion`.

To determine how well model predictions using a given σ_{RF}-θ_{RF} combination reflected empirical results, we compared model-generated velocity thresholds in a given condition – extracted from psychometric functions fitted on simulated correct responses X (for details see Methods, Early-vision model) – with the corresponding average threshold from Experiment 5 (Figure 6) and quantified their dissimilarity in terms of mean squared error (MSE).

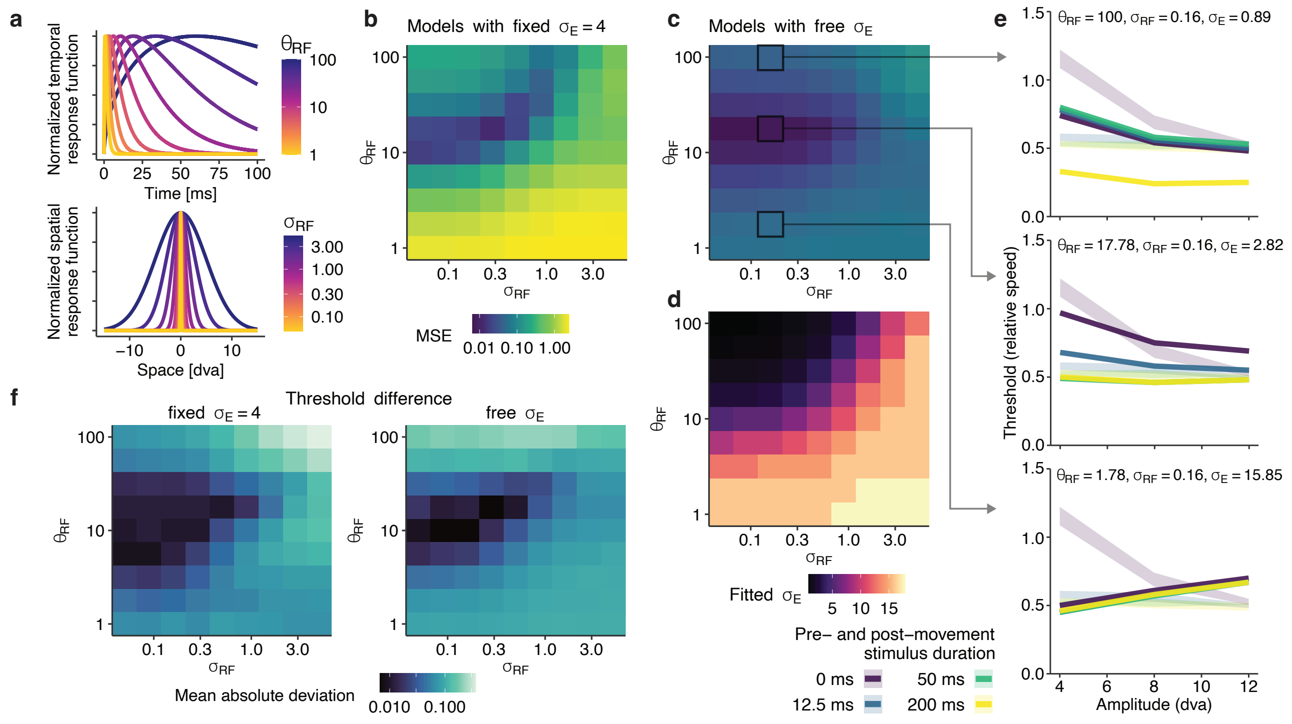

Figure S6 | Grid-search analysis to determine model parameter space. **a** Effects of changing the Gamma scale θ_{RF} and the Gaussian SD σ_{RF} on temporal (upper panel) and spatial (lower panel) response functions, respectively. **b** Results of the grid search when using a fixed SD $\sigma_E = 4$ for the Gaussian sampler applied to convert sensory evidence E to model responses X (see Methods, Early-vision model). Colors represent MSE between empirically measured velocity thresholds and velocity thresholds predicted by the model. **c** Results of the grid search in terms of MSE when an optimal σ_E is selected for each grid point. The same color scale as in panel b is used. **d** Resulting optimal σ_E for each grid point displayed in panel c. **e** Three examples of velocity thresholds predicted by the model as a function of amplitude and pre- and post-movement stimulus duration. Shaded areas represent ±2SEM around the average thresholds found in Experiment 5. **f** Mean absolute deviation of estimated thresholds for 50 ms and 200 ms of pre- and post-movement stimulus duration. Low values indicate little or no difference between velocity thresholds across movement amplitudes, that is, the signature finding in Experiment 5.

First, responses were simulated using $\sigma_E = 4$, that is, the model readout parameter used for analyses displayed in Figure 7. As shown in Figure S6b, the closest match with empirical thresholds was achieved around $\sigma_{RF} = 0.28$ cpd and $\theta_{RF} = 17.8$. These values are not only close to the ones used for the modeling in presented in Figure 7, but also physiologically plausible: Anderson & Burr (1987) estimated spatial receptive fields widths (that is, twice the SD of their Gaussian envelope) for 1 cpd to amount to 0.6 cpd, and Schweitzer *et al.* (2024) fitted post-stimulus time histograms of simple-cell activity [see 17] with the Gamma temporal response function applied here and found $k = 1.7$ and $\theta = 20.9$ to describe the data best. One may thus be tempted to conclude that the model's optimum is grounded in physiological parameters. However, this conclusion would neglect the fact that other parameter combinations could in theory produce equally good predictions, provided the amount of noise in response sampling (that is, σ_E used in converting evidence E to simulated binary responses X) were adjusted.

To this end, for each grid point, we chose the σ_E from a logarithmically scaled set (0.001 - 10,000) that minimized

MSE between model and empirical thresholds across all conditions (Figure S6c). Thereby, we controlled for the fact that evidence E would inevitably be much larger, and thus simulated velocity thresholds much higher, if less sluggish temporal response functions (i.e., lower θ_{RF}) were used. Indeed, in order to produce velocity thresholds comparable to those found during our experiments, short temporal response functions needed significantly higher σ_E, inducing larger decision noise to achieve lower velocity thresholds (Figure S6d). Whereas Figure S6c clearly shows that this procedure globally reduced MSE, grid points with $\theta_{RF} = 17.8$ and $\sigma_{RF} < 1.00$ still produced the best fits. The fact that even the smallest σ_{RF} produced good fits suggests that, in order to be able to reproduce empirical velocity-threshold patterns, the model does not strictly need a spatial kernel, especially if the Gaussian aperture of the stimulus is modeled. Inspecting individual grid-point results in Figure S6e, it becomes clear that even with best-fitting parameters (shown in middle panel), the model is not able to perfectly predict empirical thresholds, that is, especially the steep drop from lower to higher amplitudes observed without pre- and post-movement stimulus presence. The most likely explanation is that the model does not incorporate any constraints with respect to the profile of human temporal sensitivity [e.g., 18]. In other words, provided a sufficiently high temporal resolution of model, even the highest velocities (here those occurring at the largest amplitudes) would remain resolvable. As the goal of the model was to qualitatively model our results, such inaccuracies are tolerable, especially as it becomes clear that other parameter combinations, such as those displayed in top and bottom panels of Figure S6e, were clearly unable to qualitatively match empirical results.

Finally, we tested how any parameter combination in each grid point – irrespective of the quantitative difference between model predictions and empirically determined velocity thresholds – could reproduce the signature result, that is, the finding that all thresholds are similar at sufficiently long pre- and post-saccadic stimulus durations (Figure 6c,d). Specifically, the more similar thresholds are, the lower one would expect their mean absolute deviation to be. As shown in Figure S6f, largely irrespective of whether σ_E is fixed or free, it is again the parameter space previously identified where thresholds at pre- and post-saccadic stimulus durations of 50 ms and 200 ms are most similar to each other.

To conclude, even though the Early-vision model was neither intended to be fitted to the data nor set up to describe the early visual system in a appropriately detailed manner, it can still approximate the empirical data to a decent extent. How well this works greatly varies across the tested parameter space, but most critically depends on the scale parameter of the Gamma-shaped temporal response function θ_{RF}: Indeed, a physiologically plausible sluggishness of responses is needed for the model to qualitatively reproduce experimental results.

Supplementary References

1. Kuroki, Y., Nishi, T., Kobayashi, S., Oyaizu, H. & Yoshimura, S. A psychophysical study of improvements in motion-image quality by using high frame rates. *Journal of the Society for Information Display* **15,** 61 (2007).
2. Kuroki, Y. Improvement of 3D visual image quality by using high frame rate: Improvement of 3D visual image quality. *Journal of the Society for Information Display* **20,** 566–574 (2012).
3. Hoffman, D. M., Karasev, V. I. & Banks, M. S. Temporal presentation protocols in stereoscopic displays: Flicker visibility, perceived motion, and perceived depth. *Journal of the Society for Information Display* **19,** 271 (2011).
4. Emoto, M., Kusakabe, Y. & Sugawara, M. High-Frame-Rate Motion Picture Quality and Its Independence of Viewing Distance. *Journal of Display Technology* **10.** Conference Name: Journal of Display Technology, 635–641 (2014).
5. Kime, S., Galluppi, F., Lagorce, X., Benosman, R. B. & Lorenceau, J. Psychophysical Assessment of Perceptual Performance With Varying Display Frame Rates. *Journal of Display Technology* **12,** 1372–1382 (2016).
6. Khoei, M. A., Galluppi, F., Sabatier, Q., Pouget, P. & Benosman, R. Faster is better: Visual responses to motion are stronger for higher refresh rates, 22.
7. Shi, L. An Evaluation of an LCD Display With 240 Hz Frame Rate for Visual Psychophysics Experiments. *i-Perception* **8,** 2041669517736788 (2017).
8. Schweitzer, R., Doering, M., Seel, T., Raisch, J. & Rolfs, M. Saccadic omission revisited: What saccade-induced smear looks like. *bioRxiv.* eprint: `https://www.biorxiv.org/content/early/2024/09/11/2023.03.15.532538.full.pdf` (2024).
9. Van Opstal, A. & Van Gisbergen, J. Skewness of saccadic velocity profiles: A unifying parameter for normal and slow saccades. *Vision Research* **27,** 731–745 (1987).
10. Campbell, F. W. & Wurtz, R. H. Saccadic omission: why we do not see a grey-out during a saccadic eye movement. *Vision Research* **18,** 1297–1303 (1978).
11. Bouzat, S., Freije, M. L., Frapiccini, A. L. & Gasaneo, G. Inertial Movements of the Iris as the Origin of Postsaccadic Oscillations. *Physical Review Letters* **120,** 178101 (2018).
12. Schweitzer, R. & Rolfs, M. in *Eye Tracking* (ed Stuart, S.) Series Title: Neuromethods, 69–95 (Springer US, New York, NY, 2022).
13. Yee, R. D. *et al.* Velocities of vertical saccades with different eye movement recording methods. *Investigative ophthalmology & visual science* **26,** 938–944 (1985).
14. Becker, W. & Jürgens, R. Human oblique saccades: Quantitative analysis of the relation between horizontal and vertical components. *Vision Research* **30,** 893–920 (1990).
15. Zhou, W. & King, W. M. Attentional sensitivity and asymmetries of vertical saccade generation in monkey. *Vision Research* **42,** 771–779 (2002).
16. Anderson, S. J. & Burr, D. C. Receptive field size of human motion detection units. *Vision research* **27,** 621–635 (1987).
17. Reich, D. S., Mechler, F. & Victor, J. D. Temporal coding of contrast in primary visual cortex: when, what, and why. *Journal of neurophysiology* **85,** 1039–1050 (2001).
18. Kelly, D. H. Motion and vision. II. Stabilized spatio-temporal threshold surface. *Journal of the Optical Society of America* **69,** 1340–1349 (1979).
