# Supplementary material for: Lawful kinematics link eye movements to the limits of high-speed perception
Source: Nat Commun. 2025 May 8;16:3962. doi: 10.1038/s41467-025-58659-9 (PMC12062296; doi:10.1038/s41467-025-58659-9)
Supplement: Supplementary file 2 — Reporting Summary [file 41467_2025_58659_MOESM2_ESM.pdf]

nature portfolio

Corresponding author(s): Martin Rolfs

Last updated by author(s): Feb 3, 2025

Reporting Summary

Nature Portfolio wishes to improve the reproducibility of the work that we publish. This form provides structure for consistency and transparency in reporting. For further information on Nature Portfolio policies, see our Editorial Policies and the Editorial Policy Checklist.

Statistics

For all statistical analyses, confirm that the following items are present in the figure legend, table legend, main text, or Methods section.

n/a	Confirmed	
☐	☒	The exact sample size (*n*) for each experimental group/condition, given as a discrete number and unit of measurement
☐	☒	A statement on whether measurements were taken from distinct samples or whether the same sample was measured repeatedly
☐	☒	The statistical test(s) used AND whether they are one- or two-sided<br>*Only common tests should be described solely by name; describe more complex techniques in the Methods section.*
☐	☒	A description of all covariates tested
☐	☒	A description of any assumptions or corrections, such as tests of normality and adjustment for multiple comparisons
☐	☒	A full description of the statistical parameters including central tendency (e.g. means) or other basic estimates (e.g. regression coefficient) AND variation (e.g. standard deviation) or associated estimates of uncertainty (e.g. confidence intervals)
☐	☒	For null hypothesis testing, the test statistic (e.g. F, t, r) with confidence intervals, effect sizes, degrees of freedom and P value noted<br>*Give P values as exact values whenever suitable.*
☐	☒	For Bayesian analysis, information on the choice of priors and Markov chain Monte Carlo settings
☐	☒	For hierarchical and complex designs, identification of the appropriate level for tests and full reporting of outcomes
☐	☒	Estimates of effect sizes (e.g. Cohen's d, Pearson's r), indicating how they were calculated

Our web collection on statistics for biologists contains articles on many of the points above.

Software and code

Policy information about availability of computer code

Data collection	The experimental code was implemented in MATLAB (Mathworks, Natick, MA, USA), using the Psychophysics (Reference 74 in manuscript) and Eyelink toolboxes (Reference 75) running on a Dell Precision T7810 Workstation with a Debian 8 operating system.
Data analysis	The data were analysed in Matlab, using Palamedes toolbox (Version 1.11.2; Reference 36) combined with JAGS (Reference 77), and R. Analysis scripts are available on OSF (Links provided in the manuscript). Code for eyeball motion reconstruction is available here: https://github.com/richardschweitzer/PostsaccadicOscillations. Code for early vision model is available here: https://github.com/richardschweitzer/ModelingVisibilityOfSaccadelikeMotion.

For manuscripts utilizing custom algorithms or software that are central to the research but not yet described in published literature, software must be made available to editors and reviewers. We strongly encourage code deposition in a community repository (e.g. GitHub). See the Nature Portfolio guidelines for submitting code & software for further information.

Data

Policy information about availability of data

All manuscripts must include a data availability statement. This statement should provide the following information, where applicable:

- Accession codes, unique identifiers, or web links for publicly available datasets
- A description of any restrictions on data availability
- For clinical datasets or third party data, please ensure that the statement adheres to our policy

Data are available on OSF (https://osf.io/qy4dc/): Links for each experiment are provided in the Data and Code availability section of the manuscript.

Research involving human participants, their data, or biological material

Policy information about studies with human participants or human data. See also policy information about sex, gender (identity/presentation), and sexual orientation and race, ethnicity and racism.

Reporting on sex and gender	The manuscript contains data of 6 experiments from a total of 82 participants. Of these, 62 self-reported female and 20 male gender, proportions that are representative of the student population at the Department of Psychology. Other data regarding sex and gender has not been collected.
Reporting on race, ethnicity, or other socially relevant groupings	Participants were not screened for race, ethnicity, or other socially relevant groupings.
Population characteristics	All participants had normal or corrected-to-normal vision.
Recruitment	For all experiments, we recruited participants through word of mouth and campus mailing lists.
Ethics oversight	All studies were done in agreement with the Declaration of Helsinki in its latest version, approved by the Ethics Committee of the Deutsche Gesellschaft für Psychologie (Experiments 1, 3 and 4) or the Ethics board of the Department of Psychology at Humboldt-Universität zu Berlin (Experiments 2a, 2b, and 5), and pre-registered at the Open Science Framework (OSF; links provided in manuscript).

Note that full information on the approval of the study protocol must also be provided in the manuscript.

Field-specific reporting

Please select the one below that is the best fit for your research. If you are not sure, read the appropriate sections before making your selection.

☐ Life sciences ☒ Behavioural & social sciences ☐ Ecological, evolutionary & environmental sciences

For a reference copy of the document with all sections, see nature.com/documents/nr-reporting-summary-flat.pdf

Behavioural & social sciences study design

All studies must disclose on these points even when the disclosure is negative.

Study description	All five experiments, we collected qualitative data in within-subject designs.
Research sample	The manuscript contains data of 5 experiments from a total of 82 participants. Of these, 62 self-reported female and 20 male gender, proportions that are representative of the student population at the Department of Psychology. Other data has not been collected.
Sampling strategy	Except for the between-subject study (Experiment 4), we pursued a strategy in which each participant completed a large number of trials, such that each participant is expected to show the same pattern of data. We pre-registered all experiments and sample sizes. For the first experiment, we determined the sample size based on effect sizes in pilot data, which suggested that each participant's data represented a replication with remarkably similar qualitative results. All subsequent experiments used the same sample size, except for Experiments 3 and 4 which focused on correlations between eye movement kinematics and perceptual performance.
Data collection	Stimuli were projected onto a standard 16:9 (200 x 113 cm) video-projection screen (Celexon HomeCinema, Tharston, Norwich, UK), mounted on a wall in front of the participant, who rested their head on a chin rest. The high-speed PROPixx DLP projector (Vpixx Technologies, Saint-Bruno, QC, Canada) updated the visual display at 1440 Hz, with a spatial resolution of 960 x 540 pixels. Eye movements were recorded via an EyeLink 2 head-mounted system (SR Research, Osgoode, ON, Canada) at a sampling rate of 500 Hz, except in Experiments 2 and 5, in which we used an EyeLink 1000+ system at a sampling rate of 1000 Hz. Responses were collected with a standard keyboard. All data were collected by student research assistants (in a separate space) who were blind to the conditions of the experiment as well as the hypotheses.
Timing	The data were collected over several years, between 2016 and 2024, with a break in between due to a move of the lab between two campuses.
Data exclusions	Experiment 1: 3 participants had to be excluded as they did not complete all sessions.

Experiment 2a: 3 participants had to be excluded as they did not complete all sessions.
Experiment 2b: 0 participants had to be excluded.
Experiment 3: 1 participant had to be excluded as they did not complete all sessions.
Experiment 4: 4 participants had to be excluded as they did not complete all sessions.
Experiment 5: 2 participants had to be excluded; one did not complete all sessions and one performed at chance level.

Non-participation: See above, under Data exclusions.

Randomization: Participants were not allocated into experimental groups.

Reporting for specific materials, systems and methods

We require information from authors about some types of materials, experimental systems and methods used in many studies. Here, indicate whether each material, system or method listed is relevant to your study. If you are not sure if a list item applies to your research, read the appropriate section before selecting a response.

Materials & experimental systems

n/a	Involved in the study
☒	☐ Antibodies
☒	☐ Eukaryotic cell lines
☒	☐ Palaeontology and archaeology
☒	☐ Animals and other organisms
☒	☐ Clinical data
☒	☐ Dual use research of concern
☒	☐ Plants

Methods

n/a	Involved in the study
☒	☐ ChIP-seq
☒	☐ Flow cytometry
☒	☐ MRI-based neuroimaging

Plants

Seed stocks: *Report on the source of all seed stocks or other plant material used. If applicable, state the seed stock centre and catalogue number. If plant specimens were collected from the field, describe the collection location, date and sampling procedures.*

Novel plant genotypes: *Describe the methods by which all novel plant genotypes were produced. This includes those generated by transgenic approaches, gene editing, chemical/radiation-based mutagenesis and hybridization. For transgenic lines, describe the transformation method, the number of independent lines analyzed and the generation upon which experiments were performed. For gene-edited lines, describe the editor used, the endogenous sequence targeted for editing, the targeting guide RNA sequence (if applicable) and how the editor was applied.*

Authentication: *Describe any authentication procedures for each seed stock used or novel genotype generated. Describe any experiments used to assess the effect of a mutation and, where applicable, how potential secondary effects (e.g. second site T-DNA insertions, mosiacism, off-target gene editing) were examined.*
